# Complex motor task associated with non-linear BOLD responses in cerebro-cortical areas and cerebellum

**DOI:** 10.1007/s00429-015-1048-1

**Published:** 2015-04-29

**Authors:** Adnan A. S. Alahmadi, Rebecca S. Samson, David Gasston, Matteo Pardini, Karl J. Friston, Egidio D’Angelo, Ahmed T. Toosy, Claudia A. M. Wheeler-Kingshott

**Affiliations:** NMR Research Unit, Department of Neuroinflammation, Queen Square MS Centre, UCL Institute of Neurology, University College London, London, WC1N 3BG UK; Department of Diagnostic Radiology, Faculty of Applied Medical Science, King Abdulaziz University (KAU), Jeddah, Saudi Arabia; Department of Neuroimaging, Institute of Psychiatry, King’s College London, London, UK; Department of Neuroscience, Rehabilitation, Ophthalmology, Genetics, Maternal and Child Health, University of Genoa, Genoa, Italy; Wellcome Centre for Imaging Neuroscience, UCL Institute of Neurology, University College London, London, UK; Brain Connectivity Center, C. Mondino National Neurological Institute, Pavia, Italy; Department of Brain and Behavioural Sciences, University of Pavia, Pavia, Italy; Department of Brain Repair and Rehabilitation, UCL Institute of Neurology, University College London, London, UK

**Keywords:** fMRI, Force, MVC, Power grip, Visuomotor task

## Abstract

Previous studies have used fMRI to address the relationship between grip force (GF) applied to an object and BOLD response. However, whilst the majority of these studies showed a linear relationship between GF and neural activity in the contralateral M1 and ipsilateral cerebellum, animal studies have suggested the presence of non-linear components in the GF–neural activity relationship. Here, we present a methodology for assessing non-linearities in the BOLD response to different GF levels, within primary motor as well as sensory and cognitive areas and the cerebellum. To be sensitive to complex forms, we designed a feasible grip task with five GF targets using an event-related visually guided paradigm and studied a cohort of 13 healthy volunteers. Polynomial functions of increasing order were fitted to the data. Major findings: (1) activated motor areas irrespective of GF; (2) positive higher-order responses in and outside M1, involving premotor, sensory and visual areas and cerebellum; (3) negative correlations with GF, predominantly involving the visual domain. Overall, our results suggest that there are physiologically consistent behaviour patterns in cerebral and cerebellar cortices; for example, we observed the presence of a second-order effect in sensorimotor areas, consistent with an optimum metabolic response at intermediate GF levels, while higher-order behaviour was found in associative and cognitive areas. At higher GF levels, sensory-related cortical areas showed reduced activation, interpretable as a redistribution of the neural activity for more demanding tasks. These results have the potential of opening new avenues for investigating pathological mechanisms of neurological diseases.

**Electronic supplementary material:**

The online version of this article (doi:10.1007/s00429-015-1048-1) contains supplementary material, which is available to authorized users.

## Introduction

Complex motor tasks require high-level interactions and coordination between cortical areas—and between neo-cortex and cerebellum—as they depend on a host of physiological mechanisms, including sensorimotor integration, attentional processes, and performance monitoring. Thus, the evaluation of the different effects of changes in task performance on the pattern of brain responses in specific areas could provide insights into the physiological integration of different cognitive and motor functions and, potentially, their alterations in neurological conditions. Among the different techniques currently available to tackle this issue, the evaluation of the relationship between an applied hand grip force (GF), controlled by a visual cue, and the consequent blood-oxygen-level dependent (BOLD) signal modulation—as detected by functional magnetic resonance imaging (fMRI)—presents some distinctive advantages, including the availability of robust analysis techniques to assess the complex, non-linear relationship between motor performance and neural activity.

To date, the majority of studies focus on simple linear relationships between motor performance and neural responses, whilst the nature and meaning of high-order correlations between motor performance and neural activity has received significantly less attention possibly because of its difficult interpretation (e.g. Ward et al. 2007; Kuhtz-Buschbeck et al. 2008; Talelli et al. 2008). It should be noted, however, that the existence and physiological relevance of non-linear relationships between neural activity and motor performance has been described in neurophysiological literature (Ashe 1997). Indeed, in single cell recording experiments, the relation between force and neuronal activity [e.g. in primary motor cortex (M1)] was initially thought to be a simple monotonic relationship, but subsequent studies reported the presence of complex non-linear effects taking different forms and responses with no established pattern (Conrad et al. 1977; Cheney and Fetz 1980; Evarts et al. 1983; Georgopoulos et al. 1992; Maier et al. 1993; Hepp-Reymond et al. 1994; Taira et al. 1996; Ashe 1997).

Previous neuroimaging studies have mostly reported a positive linear BOLD response, with different applied GF levels, localised in the contralateral M1 and ipsilateral cerebellum, using handgrip tasks (power or precision grip) (Thickbroom et al. 1998; Dai et al. 2001; Ehrsson et al. 2001; Cramer et al. 2002; Ward et al. 2007; Kuhtz-Buschbeck et al. 2008). These studies, however, were unable to detect significant non-linear responses (where the BOLD signal exhibits a possibly non-monotonic relationship with GF), either because only two GF levels were employed or non-linearity was not explicitly investigated. To our knowledge, only three studies in healthy volunteers have found non-linear contributions to the BOLD signal in motor or non-motor areas when performing a motor task involving different GF (Ward and Frackowiak 2003; Spraker et al. 2007; Keisker et al. 2009). Reviewing the aforementioned literature, it is clear that there are inconsistent findings.

The aim of our study, therefore, was to design a non-invasive fMRI experiment that could identify and quantify, in humans, regional responses to increasing GF when performing a complex hand gripping motor task. The design was optimised to assess both linear and non-linear responses to GF variations in healthy subjects—using an event-related polynomial parametric paradigm and five GF levels. The overall hypothesis of this study was that different brain regions would show different order responses depending on their involvement (motor, sensory or cognitive) in the motor task. In particular, we performed a parametric analysis at whole brain level to assess the BOLD response both in the cerebral cortex and the cerebellum, given the latter’s role in the execution of motor functions. We also aimed to determine regions where the BOLD response would follow a second-order (positive U-shaped profile of responses, with greater activation for high and low GF, relative to intermediate levels) form, suggesting a metabolically optimum (i.e., more efficient, therefore, less metabolically demanding) response at more typical intermediate GF levels (c.f., Keisker et al. 2009). Lastly, we were interested in assessing the involvement of cognitive and associative areas in terms of their relationships with GF levels compared to motor areas. Moreover, as the paradigm involved a visual cue, we expected to find BOLD response in sensory areas, although independent of the varying GF levels.

These complex patterns once established in healthy subjects could be investigated in neurological and neurodegenerative diseases involving the motor system to disclose the degree of damage contributing to impairment.

## Materials and methods

### Subjects

Fifteen healthy volunteers with no history of neurological or psychiatric diseases [6 female, 9 male; aged 22–41 (±4.63) years] participated in this study. All subjects were right handed according to the Edinburgh handedness scaling questionnaire (Oldfield 1971). Two subjects (one male) were excluded from the study. One was excluded because their laterality index was 47 (i.e. this subject could not be classified as either right or left handed), and the other subject could not perform the task adequately (i.e. the variance of the response was very high and the subject could not maintain the performance at the highest GF levels). The mean laterality index for the remaining subjects (*N* = 13) included in the analysis was 90 (±10). All participants gave informed consent and the study was approved by the local research and ethics committee.

### MRI acquisition

A 3.0-T MRI scanner Philips Achieva system (Philips Healthcare, Best, The Netherlands) and a 32-channel receive-only head coil were used in this study. The imaging protocol comprised:

1. T1-weighted volume (3DT1): 3D inversion-recovery prepared gradient-echo (fast field echo) sequence with inversion time (TI) = 824 ms, echo time (TE)/repetition time (TR) = 3.1/6.9 ms, flip angle = 8° and voxel size = 1 mm isotropic.
2. BOLD sensitive T2*-weighted echo planner imaging (EPI): TE/TR = 35/2500 ms, voxel size = 3 × 3 × 2.7 mm^3^, inter-slice gap of 0.3 mm, SENSE factor = 2, number of slices = 46 acquired with descending order, field of view = 192 × 192 mm^2^, number of volumes = 200, number of dummy scans = 5, flip angle = 90°.

### FMRI paradigm

During BOLD acquisition, subjects performed a power grip, repetitive grip, task with their right (dominant) hand, using an MR-compatible sphygmomanometer inflation bulb (“squeeze ball”), a pneumatic flexible pad, connected to a computer suite outside the scanner room running an fMRI paradigm control system. Compression of the ball results in an air pressure measurement proportional to the force exerted—sampled at a rate of 20 Hz. The force device system is an analogue measurement recorded on an adept scientific USB-1608FS (http://directory.adeptscience.co.uk/productmcc/USB-1608FS/1/0/USB-1608FS.html) via a pressure transducer 0–1.0342e+05 pa allowing simultaneous sampling.

Each experiment comprised 75 trials divided equally into 5 GF targets (20, 30, 40, 50, and 60 % of each subject’s MVC). An event-related fMRI paradigm was developed and optimised, in terms of trial/rest timing and GF required, using the OptSeq software (http://www.surfer.nmr.mgh.harvard.edu/optseq). Each trial lasted 3 s and trials were specified in a counter-balanced and randomised order. The rest time between squeezing trials was also randomised—with a minimum of 2 s and maximum of 12 s, and comprised 55 % of the whole fMRI session (500 s).

Before the fMRI session, subjects were trained using a 2-min protocol consisting of GF levels ranging from 10 to 70 % of their MVC. The training session was divided into three parts: (1) observing an experienced person (AA) performing the task; (2) practicing the task whilst on the scanner bed but still outside the scanner bore; (3) performing the task whilst lying in the scanner bore, but without scanning. Participants lay supine on the scanner bed throughout the experiment and were instructed to extend their arms in a relaxed comfortable position. A support hand pad was provided for comfort of the arm.

**Fig. 1:**
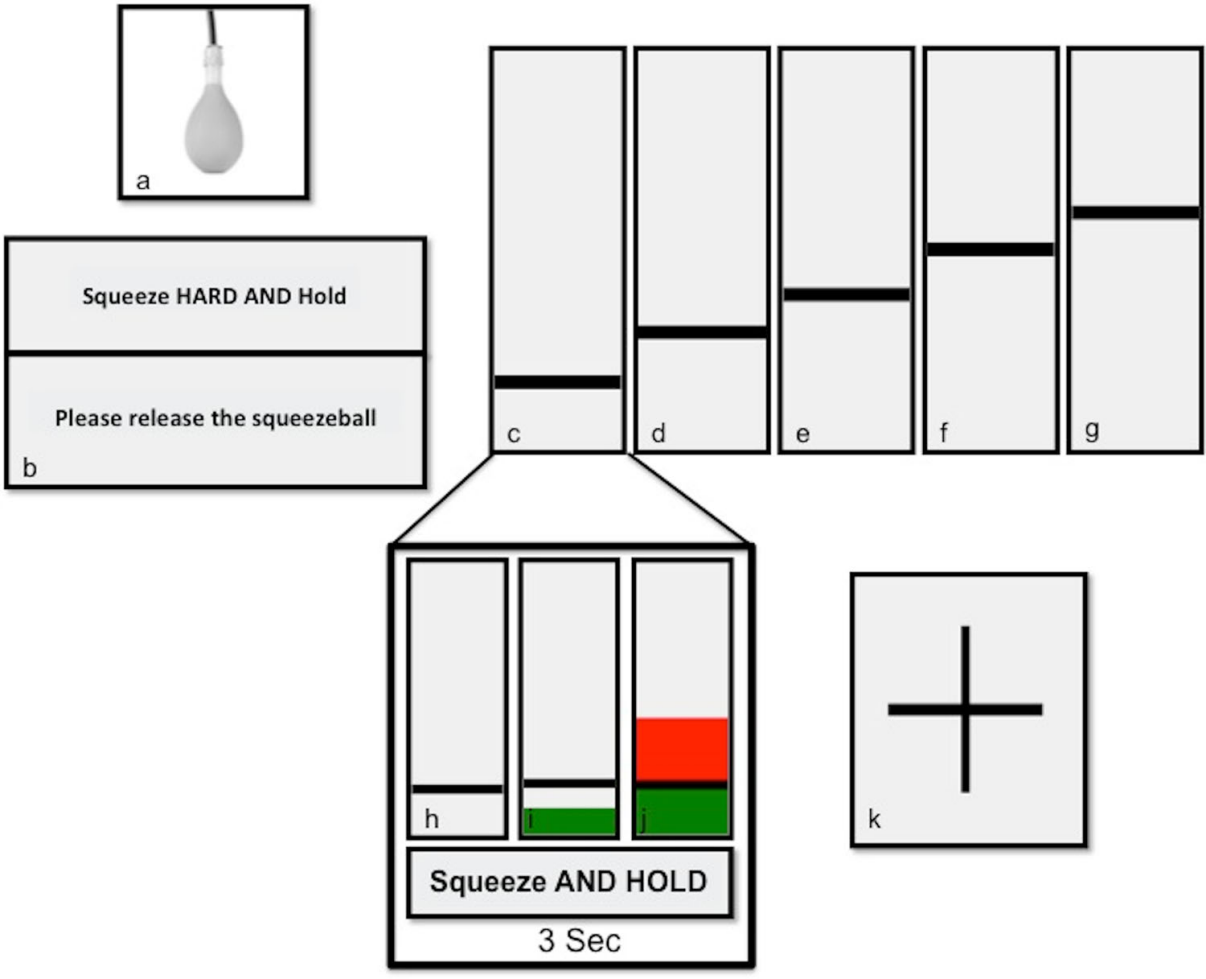
A diagram describing the steps that each subject followed. **a** The rubber flexible compatible MRI sqeezeball; **b** instructions for measuring each subject’s MVC prior to scanning; **c**–**g** the anonymous GF levels starting from 20 % with a step of 10–60 % of MVC. **h**–**j** Examples of a cued trial where **h** is the cue starting with an instructed sentence “Squeeze AND HOLD”, **i** is an example indicating that the response has not reached the required GF level while **j** shows that the response exceeds the required GF level and a *red bar* warns subjects. Lastly, **k** shows a *cross sign* indicating a rest time

The cue for the paradigm execution was implemented (by AA and DG) using Visual basic (VB.net; Microsoft, Redmond, Washington) installed on a PC running Windows XP Professional (Microsoft) and front projected onto an MR-compatible screen inside the scanner room—viewed by the subject through a mirror positioned on the head coil. MVC was first measured for each subject with the same force device (i.e. by asking the subject to apply a continuous contraction of the power ball) and used by the visual basic programme to set the GF target for each trial. The presentation consisted of two alternating images: one with a horizontal black line and one with a black crosshair located in the centre of the screen. Subjects were shown the line representing the GF target level to achieve. This was the cue to start squeezing and remained visible throughout each trial. As soon as the subject started to squeeze the ball, a green bar gave a real-time feedback indicating the applied force. Subjects were asked to try to match the height of the green bar to the position of the black line by controlling the force of their grip. Once the target was reached, the subjects had to hold it until the crosshair appeared on the screen, replacing the line and the green bar (total time per trial 3 s). If the applied force exceeded the target level, the green bar turned red to warn the subject that they were overshooting the GF requested. Figure 1 shows an example of the presentation instructions.

### Image pre-processing and analyses

Data processing was performed using statistical parametric mapping (SPM12) (http://www.fil.ion.ucl.ac.uk/spm) implemented in Matlab12b (Mathworks, Sheborn, MA). The pre-processing steps for each subject followed a standard fMRI pipeline, which includes: (i) slice time corrections performed relative to the time of acquisition of the middle slice using sinc interpolation in time (Sladky et al. 2011). This procedure is equivalent to a phase shift in the frequency domain (Sladky et al. 2011). (ii) Spatial volume realignments for motion correction performed using a least squares approach to estimate movement parameters and a six parameters (rigid body) spatial transformation. The re-slicing step was applied to the mean image only. (iii) Estimation of co-registration parameters between the re-sliced mean image (reference image) and the 3DT1 volume (source image). (iv) Estimation of (non-linear spatial) normalisation parameters between the 3DT1 volume and the standard SPM12 template. (v) Application of the normalisation parameters to the fMRI EPI volumes, resampled with a voxel size of 3 mm^3^, and (vi) smoothing of the fMRI EPI volumes with an 8 mm isotropic full-width half maximum (FWHM) Gaussian kernel.

### Statistical analysis

FMRI time series were analysed in two stages:

#### First (within subject) level

For each subject, a fixed effects analysis was performed. A parametric model (Buchel et al. 1996, 1998) was chosen to test efficiently for linear and non-linear effects. All GF values were modelled as delta functions (Friston et al. 1998). Although our (compound) trials lasted for 3 s, this duration is less than the time constant of the haemodynamic response function; the trials were, therefore, modelled as stick functions, modulated by appropriate trials-specific variables. In other words, modelling-induced responses as stick or delta functions assume that neuronal responses have a short duration in relation to the haemodynamic time constant (between 4 and 4 s). This is a standard assumption in event-related designs that has been adopted in previous grip studies (e.g. Ward et al. 2007; Boudrias et al. 2012). Parametric covariates were modelled using a set of orthogonalised polynomial expansions up to the fourth order and specified by the integral of the grip responses. We chose polynomial expansions for three reasons: first, this expansion can model a variety of linear and non-linear responses in a parsimonious fashion. For example, a simple linear effect can be estimated with a single (regression) parameter, as opposed to five separate force level parameters. Second, the interpretation of the non-linear order is more informative and lends itself to hierarchical testing. For example, the second-order effects are only interesting when one has removed first-order effects; similarly for third-order effects relative to second-order effects and so on (see Buchel et al. 1998, for more discussion). Finally, neurophysiological studies have reported different response shapes that had distinct non-linear forms (Evarts 1967; Smith et al. 1975; Conrad et al. 1977; Thach 1978; Cheney and Fetz 1980; Evarts et al. 1983; Riehle et al. 1994). The use of a high-order polynomial expansion accommodates a large family of forms with relatively few parameters. Polynomial expansions are the most common form of expansion (in the absence of boundary conditions) in estimating neurometric functions from imaging data. In particular, they have been used previously by Ward and Frackowiak 2003 and Kuhtz-Buschbeck et al. 2008.

In the polynomial expansion, the zero-order term represents the main effect of hand gripping compared to the rest condition—irrespective of the applied GF levels. The first-order expansion models any linear change with GF level; higher non-linear order modulations introduce subsequent regressors, modelling U-shaped (second-order). Also, a third-order polynomial has two points of inflection and can approximate more complicated neurometric functions—such as sigmoid functions. Modulation of the stick functions encoding grip trials with the polynomial expansion of GF produces stimulus functions that were convolved with a canonical hemodynamic response function for standard general linear model (GLM) analysis (Friston et al. 1995, 1998). The realignment parameters, from pre-processing, were also included in the GLM as regressors of no interest (Friston et al. 1996b). At this (within subject) level, *t* statistics were used to test for the effects of each polynomial coefficient. The data were high-pass filtered with a cutoff of 128 s to remove slow signal drifts and the serial correlations were accounted for in this model.

#### Second (between-subject) level

The contrast images corresponding to the five polynomial coefficients, created in the first level, were entered into a random effects analysis, testing for increasingly higher-order non-linear effects with one-sample t tests. Inferences were made at the between-subject level using the standard summary effects procedure for random effects modelling. In brief, this means summarising the response of each subject in terms of (contrasts of) the parameter estimates from the first (within-subject) models and using these as response variables for a second-level (between-subject) analysis. On average, this produces exactly the same results as a full mixed-effects analysis (Friston et al. 2005).

Cluster-level inferences (*p* < 0.05 corrected for multiple comparisons, using random field theory) were made across the whole brain (clusters were defined using an uncorrected threshold of *p* < 0.0001; minimum spatial extent ten voxels) for both analyses (Friston et al. 1996a). This represents a standard (conservative) criterion that is sensitised to locally distributed responses with a nontrivial spatial extent. Cluster peaks were anatomically designated with the SPM Anatomy toolbox (Eickhoff et al. 2005).

For the purpose of illustrating regional responses, the average modulated BOLD responses versus GF in three regions of interest (ROIs) [Brodmann area (BA) 4, 6 and 7], were calculated. In addition, we also classified or categorised the non-linear responses at each voxel (showing a significant effect) using the order of the polynomial expansion that had the greatest standardised effect size or *t* statistic. This was done separately for negative and positive responses. This characterisation does not compare different orders statistically, because differences between polynomial coefficients of different orders have no quantitative meaning (e.g. they have different units of measure). In other words, significant (non-linear) responses were identified on the basis of one or more *t* tests of polynomial coefficients being significant. The post hoc categorisation based upon the largest *t* value is simply a characterisation of standardised effect sizes that allow one to categorise response profiles: e.g. a region showing a predominant positive second-order effect will show a minimum at intermediate GF level.

### Temporal signal to noise ratio (TSNR)

Moreover, the temporal signal to noise ratio (TSNR) at the voxel level and the average TSNR map across subjects were computed. The TSNR was defined by dividing the mean of each voxel time series by its standard deviation (Hutton et al. 2011).

## Results

### Behavioural results

**Fig. 2:**
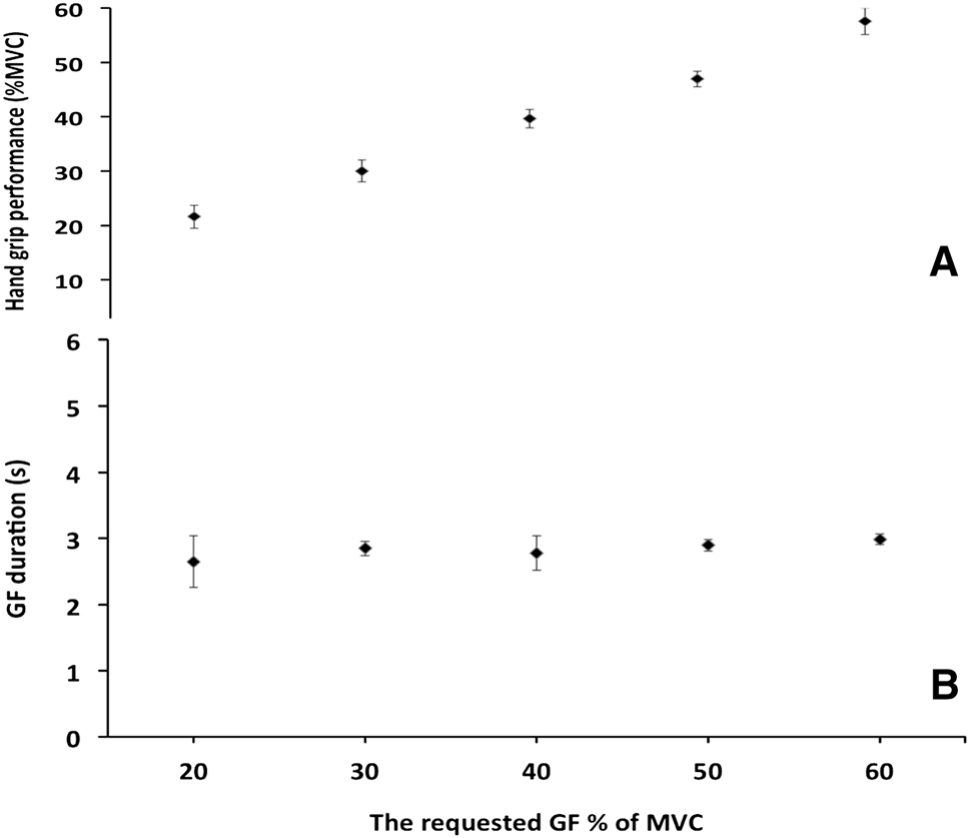
Grip performances. **a** Means of MVC ± standard deviation. (SD). Averaged MVC (±SD) was: for all trials: 39 (13), 20 %: 22 (2), 30 %: 30 (2), 40 %: 40 (2), 50 %: 47 (2), 60 %: 58 (2). **b** Means of grip duration (s) ±SD. Averaged duration (±SD) was: for all trials: 2.83 (0.32), 20 %: 2.65 (0.59), 30 %: 2.85 (0.11), 40 %: 2.78 (0.26), 50 %: 2.9 (0.09), 60 %: 2.99 (0.08)

Figure 2a, b show that all subjects were able to perform the task adequately {mean grip duration [±standard deviation (SD)] for all trails was 2.83 s (±0.3). All trials reached the requested force within 10 % of the target}.

### Within subject level example responses

**Fig. 3:**
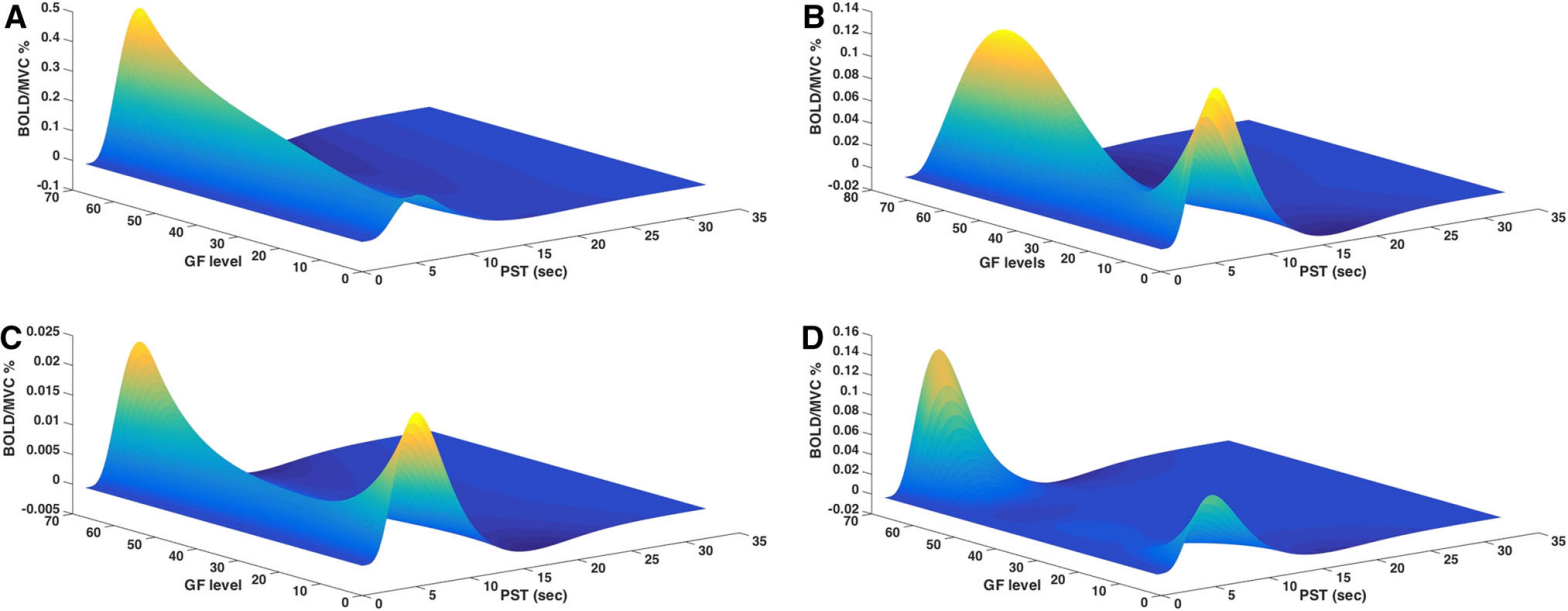
Example of BOLD responses (*Z* *axis*) of the fitted polynomial orders of GF responses (*Y axis*) at the defined post-stimulus time (PST) (*X axis*) at the subject level (fixed effect analysis). The figure shows different ROIs (**a** left BA 4a; **b** left BA 7; **c** right BA 6; **d** right BA 6) created based on the group results

Figure 3 shows examples of responses at the subject level. This figure illustrates the relationship between the GF levels and the modulated BOLD signals—and each plot represents the maximum likelihood estimates of the mapping between GF and BOLD response based on all components of the polynomial expansion.

### Group level main effects of forces (zero-order effects)

**Fig. 4:**
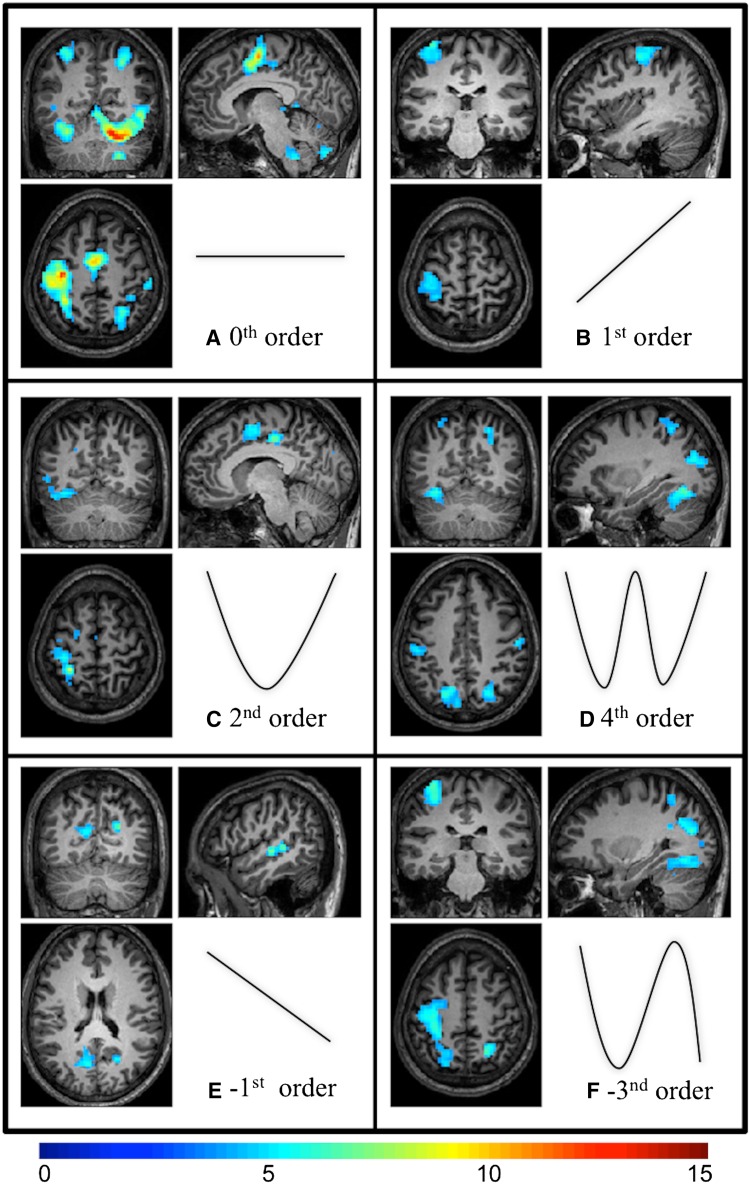
Brain activations (*T* values) at the group level corresponding to fitting polynomials of different orders to the BOLD signal response. The estimated shape of the fitted orthogonalized polynomial function is shown for each order next to the corresponding image displaying significant clusters. In the images, clusters are corrected at *p* < 0.05 after using an initial height threshold of 0.001 (for display purposes); *right* is right and *left* is left. A *T* *value colour bar* is shown below the images

The event-related design confirmed the presence of major activated motor and non-motor area networks irrespective of GF (supplementary material—Table 1; Fig. 4a). This event-related power grip task activated the contralateral M1 (BA 4 a–p), S1, bilateral cerebellum, supplementary motor area (SMA), premotor cortex, ipsilateral putamen, and some occipital (visual) areas.

### Group level effects of force on BOLD signal

#### Linear effects

Positive first-order (linear) effects were in contralateral M1 and part of the premotor cortex (BA 6) (Fig. 4b). Negative first-order (linear) effects were seen in the contralateral lingual and calcarine gyri as well as in the ipsilateral superior temporal gyrus (STG) (Fig. 4e). Supplementary material—Table 2 shows the linear polynomial coefficients in these regions.

#### Non-linear changes

Higher-order effects (2nd–4th) represent more complex associations between the BOLD response and GF. Significant non-linear associations were found in both motor and non-motor areas for positive second—(Fig. 4c), fourth—(Fig. 4d) and negative third— (Fig. 4f) order effects. Supplementary material—Table 2 reports the higher-order polynomial coefficients in these regions, including the contralateral M1/S1, cerebellum (lobule VI), cingulate cortex, fusiform gyrus (V4), bilateral SMA,  parietal and premotor cortices.

In brief, most of the 0th order networks were seen in the force-related order effects. In the force-related responses, part of the left precentral gyrus (BA 4a) was detected in the positive 1st and negative 3rd orders, while BA 4p was specific to the higher-order non-linear responses. Bilateral SMA, left middle cingulate cortex (BA 5), left postcentral gyrus, left SPL (BA 1) and right inferior frontal gyrus (BA 44) exhibited predominantly second-order effect. The positive fourth-order response predominated in areas such as left SPL (BA 7A), fusiform gyrus, and lobule VI of the cerebellum. Areas that showed a predominantly negative first-order effect are left lingual and calcarine gyri as well as right STG. Using the above threshold, the negative third-order response was predominantly seen only in the right superior occipital gyrus and SPL (BA 7A). Please refer to the supplementary material for a full list of areas, coordinates, extent of regions and *t* values.

**Fig. 5:**
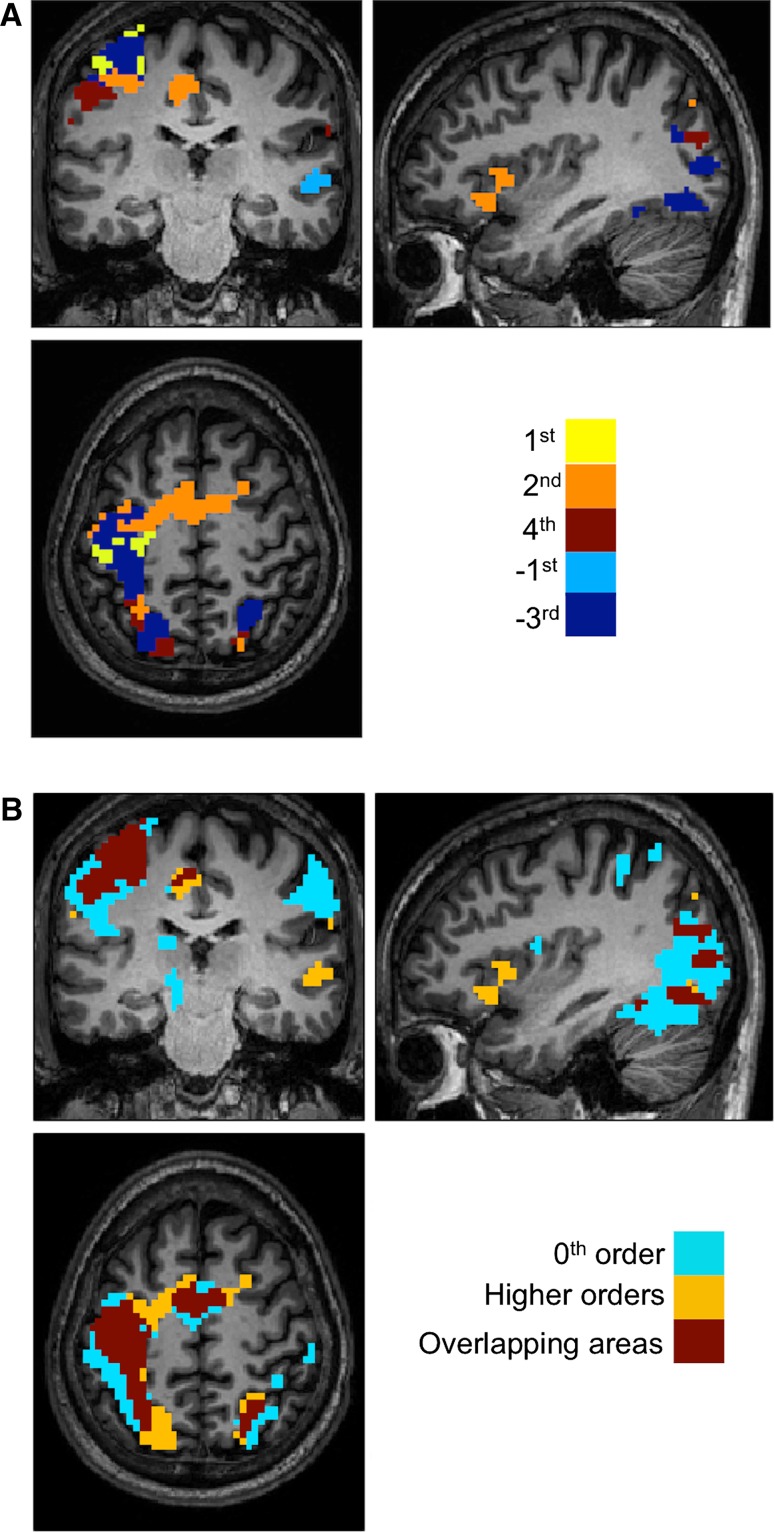
**a** Clusters of force-related effects were thresholded (using a voxel height of 0.001 and corrected (FWE) clusters) and the maximum *t* values at each voxel among all the force related orders are shown (*hot colours* are positive order responses and *cold colours* are negative responses). This is done at the group level for the purpose of illustration. In the map, *right* is right. **b** The 0th order activations, shown in *light blue*, and all force related orders, shown in *yellow*, are overlaid; overlapping areas are shown in *dark red*. The *dark red* *areas* represent the areas that are activated as a main effect of movement as well as modulated by GF levels. Note that it is not necessarily to observe a force related area that is also seen at the 0th order (e.g. *yellow areas*). In the map, *right* is right

Figure 5a, b show clusters of significant voxels that have been colour-coded based on the highest *t* value over polynomial coefficients.

### TSNR

**Fig. 6:**
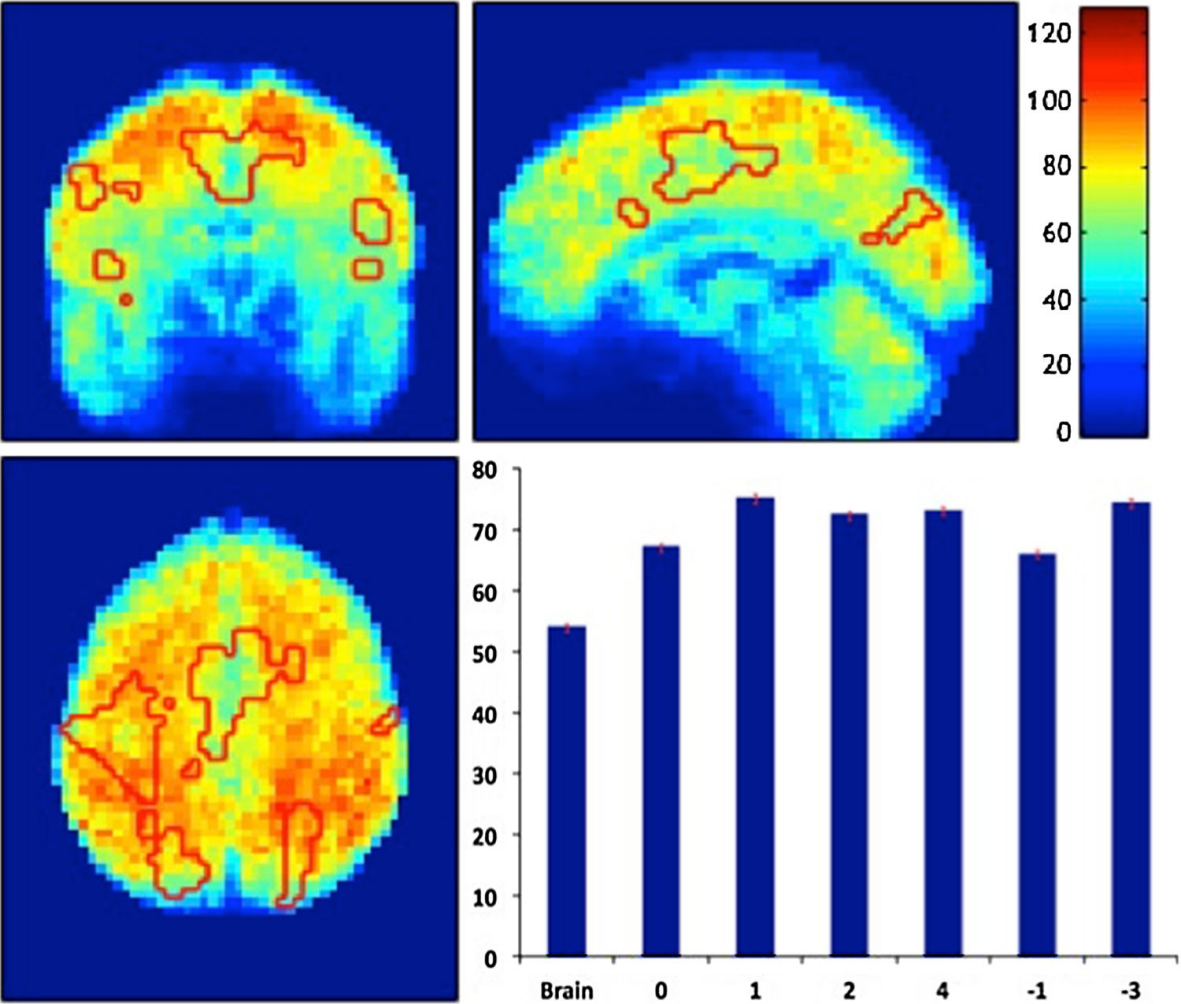
The TSNR map for the whole brain with the force-related activation areas outlined on *top* of the map (*red traces*). The average TSNR values for the whole brain and within the responded activations per order are shown in the *bar graph*. In the map, *right* is right

TSNR maps (along with the average values for the extracted clusters per order) are shown in Fig. 6. The TSNR values ranged from 40 to 120, with average values of around 70 in the extracted clusters.

## Discussion

The main aim of this study was to identify brain regions that are engaged by the grip task and to characterise their formal dependency on GF. To do this, we investigated the relationship between complex motor tasks, motor control and the BOLD signal in a group of right-handed healthy volunteers. The task, performed with the dominant hand (right) squeezing a rubber ball, also required visual and proprioceptive processing, a high degree of attention and the ability to track errors to perform the task correctly. Our main findings were that different neuronal systems contribute to task performance, with non-linear effects evident in both the cerebral cortex and the cerebellum—involving areas such as M1, SMA, premotor, sensory, parietal, visual, and cerebellar areas. Our results show: (i) categorical activation (i.e. zero order) of brain regions including the contralateral M1/S1, ipsilateral cerebellum, bilateral SMA, premotor and some parietal regions (e.g. ipsilateral SPL, contralateral IPL, contralateral postcentral gyrus) that are known to be involved in gripping motor tasks (e.g. Keisker et al. 2010), as well as additional areas such as the contralateral cerebellum, and different occipital and frontal areas; (ii) positive first-order linear effects in the contralateral M1 and premotor cortex, likely to be related to force generation; (iii) positive second- and fourth-order non-linear effects in SMA, premotor, sensory, parietal (SPL), and cerebellar (lobule VI) areas. These non-linear positive effects may be explained in terms of attention, proprioceptive movement control, visual transformation and planning or complex sequencing where a cerebral–cerebellar interaction may be key for the task execution. Also, second-order responses in key motor areas suggest that intermediate forces are metabolically optimal as they are associated with a lower BOLD response; (iv) negative first- and third-order effects preferentially localised with occipital regions linked to visual functions (e.g. BA 18), with a component in M1 and in temporal regions (e.g. STG). We believe that the reduced BOLD signal in visual areas with higher GF level could be attributed to reduced modulation of brain activity in non-motor areas during complex motor control.

The possibility to detect a complex pattern of BOLD response to a GF task has been established in this study. It is essential, therefore, to design future investigations to explore the neurophysiological basis of this behaviour with purposely designed experiments. Moreover, all these findings are opening new avenues to study the motor system and the effects of damage to the mechanism of motor function in neurological and neurodegenerative diseases such as Parkinson disease (Rickards 2005), multiple sclerosis (Mehanna and Jankovic 2013), Huntington disease (Guo et al. 2012) and spino-cerebellar ataxia (Klinke et al. 2010). Understanding the complexity of brain dynamics in response to a complex but commonly performed task in daily life may contribute also to the understanding of damage including and beyond motor areas.

Specific reports for the zero-, first- and higher-order effects and a detailed analysis of the fMRI paradigm are discussed below. Making detailed neurophysiological inferences based exclusively upon fMRI signals has some limitations, especially when trying to evaluate non-linear neurometric functions of the sort that we have tried to characterise. In particular, it must be acknowledged that non-linearities can arise at a number of levels. For example, one could be looking at non-linear neuronal responses, non-linearities in the mapping from neuronal activity to haemodynamic responses and finally, (well documented) non-linearities associated with the haemodynamic response function generating T2* signals (Friston et al. 2000; Mechelli et al. 2001).

### Main effect of force (zero order)

Our findings for zero-order effects confirm—and extend—the findings of previous power grip block fMRI studies (Cramer et al. 2002; Ward and Frackowiak 2003; Halder et al. 2007; Kuhtz-Buschbeck et al. 2008; Keisker et al. 2009, 2010; Neely et al. 2013) by showing consistent activations in motor (e.g. M1, SMA, premotor, cerebellar lobule VIII) and non-motor (e.g. BA 44, 7 and cerebellar lobule VI) areas, irrespective of GF levels. Our activated areas included M1/S1, SMA, cerebellum (lobules V–IX), parietal (including IPL and SPL), premotor cortex, basal ganglia and different frontal and visual areas (outlined in supplementary material—Table 1; Fig. 4a).

### Force-related effects

The presence of force-related effects in motor and non-motor areas has been reported in several studies (Thickbroom et al. 1998; Ehrsson et al. 2000; Dai et al. 2001; Cramer et al. 2002; Ward and Frackowiak 2003; Kuhtz-Buschbeck et al. 2008; Keisker et al. 2009). There are some differences between studies, though, in the specific reported areas. For example, Keisker et al. (2009) did not find force-related changes in the SMA nor in the cingulate cortex. This was also the case with Khutz-Buschbeck et al. (2008), whereas Ward and Frackowiak 2003 observed force-related changes in the cingulate cortex but not in the SMA. These studies used a dynamic power grip block design with different ranges of forces, from very low, 1 %, to high, 60 %. Given that we too used a range of forces up to 60 % of MVC, it may be argued that the detection of signal changes in relation to applied force in SMA may not be related to the range of GFs applied but rather to the differences in trial design, i.e., block vs. event-related. On the other hand, the range of forces may play a more significant role in detecting force-related changes in the cingulate cortex with higher GF levels up to 60 % of MVC.

Here below we will continue the discussion by commenting on the linear positive effect, non-linear positive effects and negative effects, also trying to investigate the presentation of different behaviours in different functional areas.

### Linear positive force-related effects (first order)

We found positive linear effects in contralateral M1 and premotor cortex (showed in supplementary material—Table 2; Fig. 4b). Most fMRI GF studies generally report linear effects in the contralateral M1 and ipsilateral cerebellum (Cramer et al. 2002; Ward and Frackowiak 2003; Halder et al. 2007; Ward et al. 2007; Kuhtz-Buschbeck et al. 2008; Talelli et al. 2008; Ward et al. 2008; Keisker et al. 2009). In our group of subjects, this effect is present (lobule VI), but at a lower statistical threshold (*p* < 0.003 and corrected), this may be due to the small sample size. The interpretation of a monotonic relation between BOLD response and GF levels has been related to the increased neuronal recruitment in M1 at the increase of GF (e.g. Cramer et al. and Keisker et al.).

### Non-linear positive force-related effects (second order or more)

We found higher-order (non-linear) associations between BOLD response and GF that have a clear and distributed regional specificity in both motor and non-motor areas (detailed in supplementary material—Table 2; Fig. 4c–d, f), including associative areas and the cerebellum.

#### Primary motor cortex (M1)

This study has shown clearly that in M1, in healthy subjects, there are additional positive non-linear BOLD responses induced by varying GF. Non-linear relationships in M1 have been reported in macaque monkey studies, which were interpreted as variable recruitment of neurons and/or as saturation effects (Cheney and Fetz 1980; Evarts et al. 1983; Ashe 1997). Moreover, it has been argued that within a similar cortical region, different populations of neurons may act and respond differently to direct input in a force-related task (Ashe 1997; Ward and Frackowiak 2003; Ward et al. 2006). Supporting this conjecture, Ward and Frackowiak 2003 reported different non-linear activation patterns in the right insula cortex. Similarly, in our study, within M1, different BA sub-areas (the anterior and posterior wall of the precentral gyrus represented by BA 4a and 4p, respectively) showed non-linear effects. Non-linearity in M1 may also be due to fluctuation of attention levels during the experiment as suggested by a previous fMRI study—in which neural activity within BA 4p was modulated by the degree of attention (Binkofski et al. 2002). Consistent with this finding, BA 4p responded by showing consistent non-linear effects, whereas area 4a mainly responded in a linear fashion. Furthermore, M1—and in particular BA 4p—has been reported to activate during fMRI paradigms of motor imagery (Michelon et al. 2006; Sharma et al. 2008), electroencephalography (EEG) (Lang et al. 1996; Carrillo-de-la-Pena et al. 2006) and PET (Boecker et al. 2002; Malouin et al. 2003). The finding that some regions have both linear and non-linear responses speaks to the context-sensitive nature of neuronal processing in the sensorimotor task and should be studied further to investigate whether it is possible to detect a local organisation of the microvasculature in response to a varying GF task.

Our results are consistent with the suggestion that task performance under typical force levels would produce the minimum (second-order) response. From the point of view of task set, it could be argued that maintaining GF at atypical levels is more demanding in an attentional sense. In other words, force production responses are modulated by (exogenous) attention because our task involved not only force and proprioception, but also attention and sensorimotor feedback transformations. In this context, we regard attention as an implicit component of sensorimotor integration—as opposed to endogenous attentional effects. Exogenous effects would call for a factorial design, where force level was varied independently of attentional set, for example, using a distractor task to manipulate attentional load (see below).

#### Beyond primary motor

We also found positive non-linear relationships (second and fourth orders) between GF and BOLD signal in areas outside M1. Areas included motor control and spatial attention (SMA, premotor cortex) (Macar et al. 2006; Van der Lubbe and Abrahamse 2011), associative and spatial processing visuomotor functions (SPL) (Hamzei et al. 2002; Elsinger et al. 2006), colour information processing (V4) (Coutanche and Thompson-Schill 2014), working memory and sensory-motor integration (cerebellar lobule VI) (Stoodley et al. 2010), which are areas known to be associated with complex cognitive and visuomotor tasks (Rizzolatti et al. 2002; Picard and Strick 2003; Berti et al. 2005; Kuhtz-Buschbeck et al. 2008; Nachev et al. 2008; Keisker et al. 2009; Neely et al. 2013). Keisker et al. (2009) reported non-linear components in non-primary motor areas such as S1, parietal and premotor cortices as well as in the posterior cerebellum. The profiles of the BOLD responses that Keisker et al. found mostly reflect greater BOLD response at low and high GF levels with a local minimum present with mid-force level, similar to the findings of second-order effects in our study.

BOLD response within non-M1 motor and extra-motor areas that have non-linear relationships with GF could represent the anatomical underpinnings of the complex interactions between visuomotor tasks to control visually guided movement (e.g. BA 7A) (Hamzei et al. 2002), motor performance and motor control (e.g. BA 6) (Rizzolatti et al. 2002; Berti et al. 2005), attention (e.g. BA 5), saturation, recruitment and colour processing (e.g. V4) (Coutanche and Thompson-Schill 2014). Disentangling each of these contributions is beyond the scope of this work, which intends to open a series of questions that should be addressed with purposely designed future experiments.

#### Motor control and attention areas

Controlling movement—whilst attending to proprioceptive and exteroceptive cues—to produce an accurate response during the task was a key component of our task. Although the targets were presented without an explicit indication of the force required, the levels of the targets, especially high and low, could be extrapolated by interpreting the visual cues. The non-linear response of the BOLD signal showed a dependency on the GF required in several brain regions such as SMA, premotor and cingulate cortices as well as the SPL with a corresponding decrease at intermediate (typical) GF levels. Recent neuroimaging studies showed that SMA is involved in controlling temporal processing (Macar et al. 2006) as well as controlling movement initiation and motivation for specific actions (Scangos and Stuphorn 2010). In addition, both monkey and human studies showed that the premotor cortex is involved not only in planning and execution of movements but also in spatial attention (Simon et al. 2002; Van der Lubbe and Abrahamse 2011). Therefore, these related areas (located in the BA 6) and their association with attention and movement control might explain the increased BOLD signal at the lower and higher GF levels, as more effort may be needed to attain the target level as accurately and quickly as possible. In addition, the parietal regions (e.g. SPL) are implicated in controlling movements that are visually guided—and their activity increases with increasing task complexity (Hamzei et al. 2002; Elsinger et al. 2006). A recent study showed that SPL and the visual cortex could possibly be associated with increased visuospatial processing demands in a visuomotor task such as ours, where we used a colourful feedback given by the green and red bars (Neely et al. 2013). Furthermore, the SPL receives visual input from the extrastriate visual cortex and then transfers this input to the premotor cortex. It, therefore, acts as an intermediary between the frontal and visual cortex (Marconi et al. 2001; Hamzei et al. 2002; Keisker et al. 2009). In line with this, the SPL, premotor cortex, and areas in the visual cortex (V4 and the superior occipital gyrus) showed consistent higher-order responses that may reflect the increased visual attention required in controlling low forces (Keisker et al. 2009) as well as the complexity of reaching and controlling extreme force levels. Moreover, an important finding in our study is that the premotor cortex (BA 6) as well as M1 (BA 4a and 4p) were identified by both linear and non-linear responses. As discussed in (Buch et al. 2010), a reasonable explanation is that during grasping, premotor cortex influences M1 by receiving large inputs and modulating it during reaching and grasping (Dum and Strick 2005; Prabhu et al. 2009). Since we associated BA 4a with force generation and BA 4p with attentional modulation, it is possible that area 6 controls area 4 during force production and attention, thus explaining their parallel behaviour in terms of their linear and non-linear responses.

#### Visual pathways

Visual feedback cues could play important roles in affecting cortical recruitment during motor tasks. Kuhtz-Buschbeck et al. (2008) studied main and force-related effects on BOLD signal with and without visual feedback, focusing only on two GF levels and observed more widespread-activated regions when using visual feedback. The extra regions were not only in the posterior visual pathway but also included motor pathways—and more interestingly the contralateral S1 and cerebellum as well as the ipsilateral parietal region. Since subjects saw their actual grip responses, as in this study, this could indicate the involvement of imaginary recruitments of motor areas (Mizuguchi et al. 2013), due to the usage of real feedback signals, as well as gaining awareness (de Graaf et al. 2004). Another explanation of the contribution of the visual feedback signal in our non-linear observations could be error tracking (Imamizu et al. 2000; Milner et al. 2007) to try and maintain an accurate production of force whilst watching the actual response. Our task included visual feedback signal where subjects saw their performance in real time and adjusted their applied force to match the target. It has, in fact, been shown that the percentage signal change in the precentral gyrus is not significantly different between low and high GF levels when using a visual cue. On the other hand, the same region exhibited a significant signal increase at the higher GF, in the absence of the visual cue (Noble et al. 2013).

#### The cerebellum

In our study, we observed recruitment of a large area of the ipsilateral cerebellum (lobules V-IX) as well the involvement of a contralateral region (lobules VI and VIIIa) showing zero and higher-order behaviour. Lobule VI was previously found to be involved in a working memory task by (Stoodley et al. 2010) and could have a role in interpreting the visual feedback involved in our task. One could hypothesise that the cerebellum is key to error tracking and forward control (D’Angelo and Casali 2013); therefore mediates the ability of the subjects to maintain an accurate performance even at low and high GF levels where error detection must require greater effort.

#### Associative areas and mirror neurons

Some of the complex non-linear relations between the BOLD response and GF seem to involve parietal areas (BA 5), which have been associated with the presence of mirror neurons; i.e., visual–motor neurons activated when observing motor tasks performed by others (Molenberghs et al. 2012) or observing an experiment (Calvo-Merino et al. 2005). Further experiments to investigate this hypothesis using action observation of the force grip task used in this study are warranted.

### Negative force-related effects (first and third order)

Negative linear responses in different brain regions have been reported in other fMRI studies (Ehrsson et al. 2001; Ward and Frackowiak 2003; Kuhtz-Buschbeck et al. 2008; Talelli et al. 2008; van Duinen et al. 2008; and our study) but there seem to be no consistent findings. Regions included contralateral angular gyrus, bilateral premotor areas, SMA, and ipsilateral parietal regions (Kuhtz-Buschbeck et al. 2008). One study, instead, did not detect negative linear effects at all (Ward et al. 2007).

An interesting observation of our study is that apart from two clusters in the primary motor cortex, all areas showing a negative BOLD correlation with GF level are known to be involved in visual functions. One possible explanation is that areas engaging with visual tasks become subordinate in terms of BOLD response compared to associative and motor control areas as the GF required increases, suggesting a redistribution of oxygen demand. It is also possible that this behaviour, in particular in M1 (BA 4) may imply some degree of fatigue at higher GF levels, already reported by others where motor fatigue was associated with diminished activity in M1, S1, SMA and different frontal visual areas (Benwell et al. 2005, 2006, 2007; van Duinen et al. 2007). It could also indicate the ability of M1 to address energy demand with a very fine localised microvasculature reorganisation.

### The fMRI paradigm

We used a polynomial parametric event-related design to test for the relationship between variable GF levels and BOLD signal in healthy volunteers—with the aim of identifying and quantifying non-linear effects. To the best of our knowledge, this is the first study that has used such a design to characterise the higher order non-linear effects of complex motor tasks on the BOLD signal. The main reasons for using an event-related design in this study were to avoid performance variability, and make the task effort consistent across subjects, thus allowing accurate measurements of GF responses. We used a dynamic power grip task as opposed to a precision or static task since it is physiologically more relevant to every-day human life, and has been a valuable tool in investigating different motor impairment diseases such as stroke (Ward et al. 2003) and multiple sclerosis (White et al. 2009) as well as investigating age-related changes (Park et al. 2012). GF levels varied between 20 and 60 % of each subject’s MVC, in steps of 10 %. We chose this range for three reasons: first, it is the approximated average of most previous fMRI GF studies (Ward and Frackowiak 2003; Pope et al. 2005; Spraker et al. 2007; van Duinen et al. 2007; Ward et al. 2007; Kuhtz-Buschbeck et al. 2008; Talelli et al. 2008; Keisker et al. 2009; Sterr et al. 2009; Boudrias et al. 2012); second, we aimed to introduce a large range between the lower and higher GF levels over five steps to be sensitive to higher-order non-linear relations with the BOLD signal; thirdly, different daily common functional tasks require a range of different force levels between 20 and 60 % MVC (Marshall and Armstrong 2004). We also used fifteen trials per force, which was chosen as the result of an optimization process that found a compromise between the scanning time and the design efficiency. This choice was also supported by the fact that previous studies showed that it is possible to detect motor activations with event designs using an even lower number of trials in healthy subjects and in patients (Ward et al. 2006, 2007, 2008). Fixed 3-s stimulation durations, reflecting a dynamic grip task (King et al. 2014), were used to enforce a consistent and transient grip pattern across trials—as it has been shown there are specific networks controlling different movement patterns (static or dynamic) (Keisker et al. 2010; King et al. 2014). Fixed grip duration has been used widely in motor fMRI studies (e.g.Ward and Frackowiak 2003; Kuhtz-Buschbeck et al. 2008; Keisker et al. 2009). Furthermore, the randomised counterbalanced event design ensured that the task requirements were unpredictable (see Fig. 1). Given the brief duration of GF changes and its unpredictability, we assume (to a first approximation) that habituation would be minimal and would, therefore, not depend on the applied force levels—or confound neuronal responses. Furthermore subjects underwent a training period prior to the actual examination, while already on the MRI table, which limited possible performance anxiety issues during the task itself.

Moreover, the training and instruction session before the actual fMRI run aimed to minimise errors in motor performance during the task and, at the same time, limit the influence of learning (Spraker et al. 2012). Lastly, the inter-subject variability in task performance (shown in Fig. 2a, b) was very small—and overall the task was performed with a good reproducibility. Also, the calculated TSNR, shown in Fig. 6, gives typical TSNR across the brain in other studies that used a similar protocol and scan strength (Murphy et al. 2007; Gonzalez-Castillo et al. 2011; Hutton et al. 2011). Therefore, it is unlikely that regional variations in signal to noise have differentially affected a sensitivity to high- or low order GF effects.

### Methodological considerations

The partial agreement of our findings with Ward and Frackowiak (2003) and Keisker et al. (2009) and the differences with previous studies could be related to three methodological considerations affecting the BOLD signal and its relation with force scaling: (1) the timings of the experimental task and design, (2) the number and range of the targeted forces, and (3) the guided cue to perform the task: (1) The relationship between BOLD and GF has been mostly studied using a power GF task (repetitive pulses) (Cramer et al. 2002; Ward and Frackowiak 2003; Halder et al. 2007; Ward et al. 2007; Kuhtz-Buschbeck et al. 2008; Keisker et al. 2009; Neely et al. 2013) or a static force task (sustaining the force) (Dai et al. 2001; Keisker et al. 2010; Neely et al. 2013). Comparing these two tasks, it is evident that they reveal different brain network regions (Keisker et al. 2010; Neely et al. 2013; King et al. 2014). The experimental paradigm can be basic and simple using a block design (e.g. Sterr et al. 2009), effortless using a sparse event-related design (e.g. Ward et al. 2006), or complex and challenging using a rapid event-related design. Assuming that the aim is to quantify the relationship between GF and BOLD signal, one would need to use a design that accurately reflects the subject’s performance and allows an efficient estimation of force-related responses. Although event-related designs are less efficient in statistical terms at detecting BOLD responses, they nevertheless allow detailed sampling of the GF levels applied during the task and are less prone to fatigue than the theoretically more efficient block designs. (2) Most of the previous aforementioned studies (e.g. Kuhtz-Buschbeck et al. 2008; Keisker et al. 2009) estimated the averaged forces over several seconds (over 10 s) due to the use of block designs and averaging GF responses over a number of epochs, which is usually limited to be around five. The number of GF levels in the previous experiments was mostly three, with the highest being five (e.g. Spraker et al. 2007) and the lowest being two (e.g. Pope et al. 2005). Two GF levels can only reveal a linear relationship. Ideally, one would need to expand the range of forces to accurately test for the relationship between BOLD and force. Five targets, as in our design, was a compromise between this requirement and the need to sample enough data for efficient statistical analysis of the signal changes. The range of forces is also an important factor that could affect the recruitment of areas. For instance, low forces seem to recruit (different, fewer or additional) areas as compared to high forces (Ehrsson et al. 2001; Ward and Frackowiak 2003; Kuhtz-Buschbeck et al. 2008; van Duinen et al. 2008; Keisker et al. 2009). Lastly, (3) the guided cue, as discussed before, plays an important factor and could also determine brain activations. The most widely used external cues for this type of experiment are visual (Sterr et al. 2009), which have been shown to activate additional brain networks (Kuhtz-Buschbeck et al. 2008; Noble et al. 2013).

In addition, this study has its own limitations. The first limitation is related to the use of a system composed of an elastic squeeze ball that relates the applied force to air pressure measurements. Although a similar equipment set-up has been used previously (e.g. Halder et al. 2005, 2007; Schmidt et al. 2009; Kurniawan et al. 2010), it is not necessarily optimal. For example, the visco-elastic properties of the squeeze ball material itself could have contributed to the non-linear effects. Despite this potential confounding effect, all volunteers who participated in this study performed the task equally well; therefore, the measured response was able to track the requested force (Fig. 2a). Future studies may want to consider alternative experimental set-ups [e.g. water filled systems (Noble et al. 2011, 2013)]. This present study assumed that these potentially confounding effects were small in relation to the effect sizes of the physiologically mediated processes. Finally, a limitation of this study is the absence of a quantitative neurophysiological measurement to support the interpretation of the nature of the non-linear parametric responses. Future similar studies should include electrophysiology measurements and arterial spin labelling to isolate the precise contributions of neuronal, haemodynamic, and BOLD biophysics to the non-linear effects characterised in this study.

## Conclusions

This study has shown that it is possible to characterise non-linear contributions to motor task performance using an optimised acquisition and analysis protocol, based on event-related design, which complements the widespread use of block design. We have demonstrated linear and non-linear responses in M1 using five GF levels, event-related paradigm, and polynomial function. Interestingly, non-linear responses in M1, especially the posterior part of M1 (BA 4p), have been associated with attention and motor imagery, which may be involved in executing our event-related grasping task. Associative cortical areas and cerebellar lobule VI are involved in higher-order effects, indicating their recruitment during coordination between visual cues, sensorimotor feedback and error tracking. We have also shown that the premotor, SMA and parietal regions, known to participate in movement control and attention, show zero-order as well as higher-order BOLD effects. The low BOLD response associated with intermediate GF levels shown with the second-order analysis is suggestive of an optimum metabolic response in key motor areas. Finally, the use of visual feedback in motor paradigms is likely to have added a layer of complexity, especially when interpreting negative non-linear responses. The strong evidence of a complex pattern of responses warrants further studies that will need to be designed with the aim of explaining the specific physiological correlates of the dissociable effects that our parametric study has revealed.

## Electronic supplementary material

Below is the link to the electronic supplementary material.

Supplementary material 1 (DOCX 98 kb)
