# Supplementary material for: Complex motor task associated with non-linear BOLD responses in cerebro-cortical areas and cerebellum
Source: Brain Struct Funct. 2015 Apr 29;221:2443–58. doi: 10.1007/s00429-015-1048-1 (PMC4884204; doi:10.1007/s00429-015-1048-1)
Supplement: Supplementary file 1 — Supplementary material 1 (DOCX 98 kb) [file 429_2015_1048_MOESM1_ESM.docx]

| **Table 1** Brain regions that are activated for zero order effects of force. This shows the main effect of hand gripping irrespective of force level. | | | | | | | |
| --- | --- | --- | --- | --- | --- | --- | --- |
|  | | | | | | | |
|  | |  | **Coordinates (MNI)** | | |  | |
| **P(cor)** | **KE** | **T** | **x** | **y** | **z** | **Anatomical region** | **BA/Loc (%)** |
| **0 order** |  |  |  |  |  |  |  |
| 0.000 | 894 | 13.76 | 27 | -52 | -29 | Right Cerebellum* | Lobule VI (85) |
|  |  | 13.38 | 18 | -55 | -26 | Right Cerebellum* | Lobule VI (70) |
| 0.000 | 880 | 13.56 | -45 | -31 | 43 | Left Inferior Parietal Lobule* | 2(70) |
|  |  | 12.00 | -36 | -19 | 52 | Left Precentral Gyrus* | 4p/4a (40) |
|  |  | 11.57 | -51 | -22 | 31 | Left Postcentral Gyrus* | IPC (PFop) (50) |
| 0.000 | 133 | 12.03 | 21 | -64 | -50 | Right Cerebellum* | Lobule VIIIa (55) |
|  |  | 8.38 | 15 | -46 | -50 | Right Cerebellum* | Lobule IX (66) |
|  |  | 6.47 | 27 | -49 | -50 | Right Cerebellum | Lobule VIIIb (46) |
| 0.000 | 216 | 11.42 | -3 | -7 | 55 | Left SMA | 6(70) |
|  |  | 5.92 | 12 | 2 | 46 | Right SMA | 6(30) |
| 0.009 | 25 | 9.61 | 45 | -4 | 10 | Right Rolandic Operculum* | OP 3 (20) |
|  |  | 8.11 | 54 | 8 | 4 | Right Rolandic Operculum | 44 (40) |
| 0.000 | 113 | 9.39 | 60 | -25 | 34 | Right Supra Marginal Gyrus* | IPC (PFt) (50) |
|  |  | 7.46 | 48 | -31 | 37 | Right Supra Marginal Gyrus | IPC (PFt), hIP2 (30) |
|  |  | 6.87 | 57 | -13 | 25 | Right Supra Marginal Gyrus | IPC (PFop), 3b (30) |
| 0.000 | 287 | 8.99 | -45 | -67 | -2 | Left Middle Occipital Gyrus* | hOC5 (V5) (20) |
|  |  | 7.86 | -30 | -55 | -23 | Left Cerebellum | Lobule VI (100) |
|  |  | 7.15 | -36 | -55 | -17 | Left Fusiform Gyrus |  |
| 0.001 | 46 | 8.14 | -15 | -70 | -44 | Left Cerebellum | Lobule VIIIa (34) |
| 0.000 | 82 | 7.83 | 60 | 11 | 25 | Right Inferior Frontal Gyrus p. Opercularis | 44 (40) |
|  |  | 7.11 | 51 | 8 | 28 | Right Inferior Frontal Gyrus p. Opercularis | 44 (40) |
|  |  | 6.89 | 57 | 5 | 37 | Right Precentral Gyrus | 6 (60) |
| 0.037 | 16 | 7.45 | 27 | -1 | -2 | Right Putamen |  |
|  |  | 6.38 | 24 | 8 | -8 | Right Putamen |  |
|  |  | 5.52 | 24 | 5 | 4 | Right Putamen |  |
| 0.002 | 35 | 7.02 | 33 | -58 | 58 | Right Superior Parietal Lobule | SPL (7A) (60) |
|  |  | 6.97 | 27 | -55 | 49 | Right Superior Parietal Lobule | hIP3 (40) |
| 0.005 | 30 | 6.67 | -57 | 5 | 37 | Left Precentral Gyrus | 6 (60) |
|  |  | 6.07 | -57 | 11 | 22 | Left Inferior Frontal Gyrus p. Opercularis | 44 (30) |
| Regions are corrected (P<0.05 (FWE) at the cluster level). *corrected P<0.05 at the voxel level. The last column on the right shows the probability (%) of these voxels to be located in the respective Brodmann areas (BA) or specified location (Loc) according to the cytoarchitectonic maps. P(cor)= P-value corrected at the cluster level; KE= number of voxels in a cluster; T= T-value at the voxel level. | | | | | | | |

| **Table 2** Force related effects on BOLD signal reported for the five polynomial coefficients which resulted in significant clusters. | | | | | | | |
| --- | --- | --- | --- | --- | --- | --- | --- |
|  | | | | | |  | |
|  | |  | **Coordinates (MNI)** | | |  | |
| **P(cor)** | **KE** | **T** | **x** | **y** | **z** | **Anatomical region** | **BA/Loc (%)** |
| ***1 order*** |  |  |  |  |  |  | |
| 0.024 | 16 | 6.67 | -36 | -28 | 67 | Left Precentral Gyrus | 6, 4a (50) |
| 0.005 | 25 | 6.06 | -39 | -19 | 55 | Left Precentral Gyrus | 4a (60) |
| ***2 order*** |  |  |  |  |  |  |  |
| 0.018 | 20 | 10.13 | -27 | -43 | 64 | Left Superior Parietal Lobule* | 1 (50) |
| 0.011 | 23 | 7.91 | 12 | -1 | 55 | Right SMA | 6 (30) |
|  |  | 5.93 | 21 | 5 | 49 | Right area 6 | 6 (10) |
| 0.000 | 50 | 7.12 | 0 | -1 | 40 | Left Middle Cingulate Cortex | 6 (20) |
|  |  | 6.14 | -12 | 2 | 55 | Left area 6 | 6 (40) |
|  |  | 5.89 | -3 | -7 | 49 | Left Middle Cingulate Cortex | 6 (80) |
| 0.011 | 23 | 7.06 | -9 | -22 | 43 | Left Middle Cingulate Cortex | SPL (5Ci) (20) |
| 0.000 | 60 | 6.97 | 51 | 8 | 28 | Right Inferior Frontal Gyrus p. Opercularis | 44 (40) |
| 0.004 | 31 | 6.59 | -30 | -22 | 46 | Left Area 4p | 4p, 3a (40) |
|  |  | 5.82 | -36 | -13 | 55 | Left Precentral Gyrus | 6 (70) |
|  |  | 5.67 | -51 | -20 | 30 | Left Postcentral Gyrus |  |
| ***4 order*** |  |  |  |  |  |  |  |
| 0.004 | 34 | 6.85 | -15 | -67 | 46 | Left Superior Parietal Lobule | SPL (7A) (30) |
| 0.001 | 47 | 6.80 | -33 | -67 | -14 | Left Fusiform Gyrus | hOC4v (V4) |
|  |  | 5.80 | -33 | -52 | -23 | Left Cerebellum | Lobule VI (68) |
| ***-1 order*** |  |  |  |  |  |  |  |
| 0.035 | 14 | 8.21 | -18 | -52 | -2 | Left Lingual Gyrus | 18 (50) |
| 0.001 | 35 | 7.25 | 54 | -22 | -2 | Right Superior Temporal Gyrus |  |
|  |  | 6.92 | 51 | -31 | 4 | Right Superior Temporal Gyrus |  |
| 0.011 | 20 | 6.92 | -6 | -70 | 22 | Left Calcarine Gyrus | 18 (30) |
| ***-3 order*** |  |  |  |  |  |  |  |
| 0.000 | 153 | 7.67 | -36 | -28 | 64 | Left Precentral Gyrus | 4a (50) |
|  |  | 6.09 | -33 | -19 | 52 | Left Precentral Gyrus | 4p (30) |
| 0.027 | 21 | 6.53 | 30 | -73 | 19 | Right Superior Occipital Gyrus |  |
|  |  | 5.46 | 30 | -61 | 28 |  | SPL (7A) (10) |
| Regions are corrected (P<0.05 (FWE) at the cluster level). *corrected P<0.05 at the voxel level. The last column on the right shows the probability (%) of these voxels to be located in the respective Brodmann areas (BA) or specified location (Loc) according to the cytoarchitectonic maps. P(cor)= P-value corrected at the cluster level; KE= number of voxels in a cluster; T= T-value. | | | | | | | |
